# Editorial: The Role of Platelets in Cancer Progression and Malignancy

**DOI:** 10.3389/fonc.2021.814006

**Published:** 2021-12-15

**Authors:** Martin Schlesinger, Martina Gobec, Alexander T. Bauer

**Affiliations:** ^1^ Pharmaceutical Institute, University of Bonn, Bonn, Germany; ^2^ Faculty of Pharmacy, University of Ljubljana, Ljubljana, Slovenia; ^3^ Department of Dermatology and Venereology, University Hospital Hamburg-Eppendorf, Hamburg, Germany

**Keywords:** platelets, cancer, metastasis, thrombosis, inflammation, biomarkers, therapeutic targets

The association between malignancy and coagulation is known since more than 150 years and was initially described by Armand Trousseau (1). Thrombotic complications are not only a diagnostic hind for an occult cancer (2), venous thromboembolism is also the second leading cause of death in cancer patients (3). Although novel treatment strategies, such as immune checkpoint inhibition or CAR T cell therapy have revolutionized cancer therapy and have drastically improved the prognosis for some tumor entities, a high incidence of thrombotic events is recognized (4, 5). The same studies clearly identify a correlation between thrombosis, treatment side effects and an increased mortality. In this context, platelets are known for their crucial roles linking coagulation, metastasis and the local immune regulation (6). For instance, platelets secrete proinflammatory cytokines and adhere to leukocytes (7). When interacting with T cells, platelets can selectively modulate T cell activation and recruitment (8), and are also able to impact the function of further leukocyte populations (9). However, although best recognized for their roles in hemostasis and inflammation, the underlying mechanisms of this complex interplay in malignancy are poorly understood.

In this Research Topic on the role of platelets in cancer progression and malignancy, we compiled three reviews and two research articles. The reviews address the impact of platelets on liver disease and cancer, platelet-tumor cell interactions in the different steps of the metastatic cascade and the underlying molecular mechanisms. Two original research articles evaluate the role of platelets as potential biomarkers for breast cancer progression and the impact of platelets in thrombosis and tumor growth in pancreatic tumors.

In the past, several reports underline the relevance of platelets for the pathophysiology of the liver (10). Marie et al. present findings on the crucial role of platelets in the course of liver disease. The current knowledge of how liver injury results in a dysregulated coagulation and alters platelet function is discussed. Vice versa, it was recently shown that platelets contribute to the conversion of non-alcoholic steatohepatitis to liver cirrhosis and ultimately hepatocellular carcinoma (11). Platelet adhesion, platelet activation and granule secretion were identified as pivotal for hepatocarcinogenesis. Significantly, analogous mechanisms are involved in the development of metastasis. A special focus on the impact of platelets in the different steps of metastasis formation and how circulating platelets orchestrate cancer cell invasion, intravasation, the survival of tumor cells in the circulation and extravasation is given by the mini review of Fabricius et al. Particularly, platelet-derived P-selectin is highlighted and preclinical evidence demonstrated that the binding of platelets *via* P-selectin not only protects circulating tumor cells in the blood stream but also promotes tumor cell extravasation. However, a critical view on the experimental data imply fundamental species-specific differences between murine and human platelets in P-selectin levels and binding to their ligands on cancer cells. This could be a plausible explanation for the largely unrealized potential of anti-platelet therapies in malignancy and many mechanistic questions remain to be answered to ensure future clinical applications. With similar motivation in mind, Braun et al., offer an extended literary overview of the molecular mechanisms of tumor cell-platelet interactions. This review article advances our understanding about the role of platelets in cancer by presenting relevant contributions of platelets to cancer-associated thrombosis and cancer-associated inflammation. Circulating platelets are equipped with a vast amount of molecules involved in metastasis and thrombosis, e. g. VEGF-A (12), P-selectin (13), VWF (14) and platelet factor 4 (15). Consequently, the identification of molecules associated with activated platelets represent promising candidates to serve as predictive and prognostic clinical biomarkers. The group of Hinterleitner et al. addresses the potential use of the platelet-derived Tumor necrosis factor Receptor TNFRSF13B (TACl) as biomarker in breast cancer patients. Using immunofluorescent analysis and flow cytometry the authors provide clear evidence of an increased TACl expression on the surface of platelets isolated of breast cancer patients when compared to healthy donors. Interestingly, platelet TACl inversely correlated to the disease stages. Patients with metastases exhibited less platelet expression than patients with a localized primary tumor. Thus, TACl on platelets may have great clinical potential in predicting breast cancer and seems to provide important predictive value for the occurrence of metastasis. Based on the critical role of platelets in tumor progression, and the existence of abundant literature on anti-platelet strategies in malignancy, one may speculate that anti-platelet therapies could be promising for cancer patients (16). Although preclinical data show interesting results using inhibitors for purinergic ADP receptors on the platelet surface (17), the results from clinical trials are conflicting. Treatment with clopridrogel even increased the incidence for the development of cancer and the risk of tumor-related mortality (16, 18). This highlights the urgent need for further mechanistic studies for a better understanding of molecular mechanisms of platelet inhibition in the context of cancer and thrombosis. Finally, this intention has been taken up by the experimental study of Palacios-Acedo et al. Using a combination of *in vitro* systems and a mouse model for pancreatic cancers, the authors make the important observation that compared to aspirin, clopidrogel exhibited a superior therapeutic benefit by inhibition of the proliferation of pancreatic adenocarcinoma cells. Importantly, work in preclinical models for malignant melanoma have shown that platelets suppress T cell functions and emphasize the validity of aspririn and clopidrogel for combinations with immunotherapeutics (19).

Overall, the collection of articles in this Research Topic highlights platelets as important link between thrombosis, treatment side effects and tumor progression. Hence, a deepening of our understanding of cancer-related coagulation and inflammation with regard to platelet functions will accelerate the design of novel treatment strategies to target thrombosis and metastasis in the future (**Figure 1**). 

**Figure 1:**
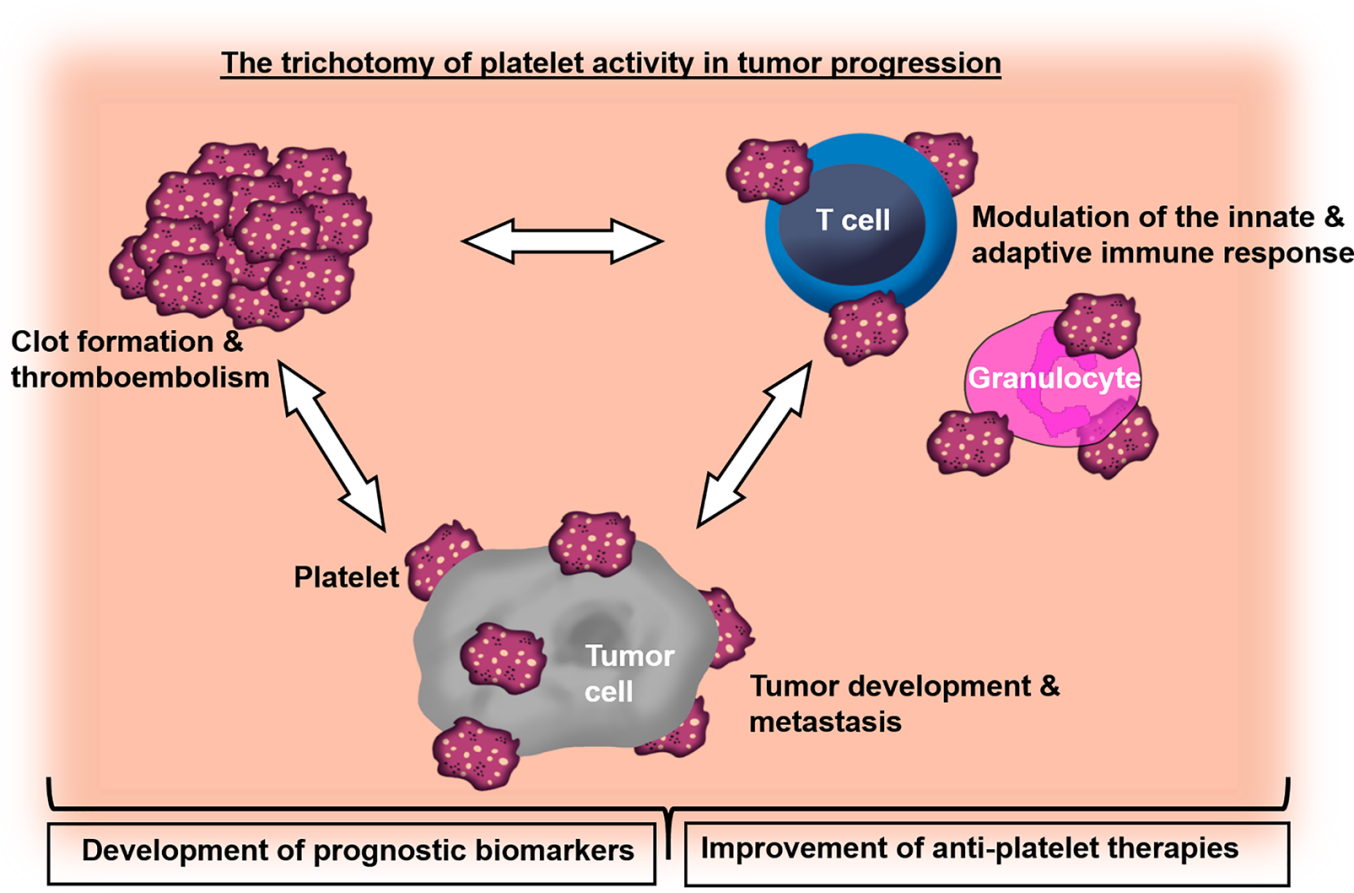
Graphical overview demonstrating the role of platelets in the crosstalk of cancer-associated thrombosis, inflammation and tumor progression.

## Author Contributions

All authors listed have provided a substantial, direct and intellectual contribution to the manuscript and have approved it before publication.

## Conflict of Interest

The authors declare that the research was conducted in the absence of any commercial or financial relationships that could be construed as a potential conflict of interest.

## Publisher’s Note

All claims expressed in this article are solely those of the authors and do not necessarily represent those of their affiliated organizations, or those of the publisher, the editors and the reviewers. Any product that may be evaluated in this article, or claim that may be made by its manufacturer, is not guaranteed or endorsed by the publisher.
